# Reversible Cerebral Vasoconstriction Syndrome and Ischemic Stroke Secondary to Peripartum Cardiomyopathy - Report a Rare Case

**DOI:** 10.7759/cureus.29640

**Published:** 2022-09-27

**Authors:** Stephanie Hang, Priyadarshini Dixit, Dilnaz Alam, Sarah Fatima, Ramesh Madhavan

**Affiliations:** 1 Internal Medicine, Saint Joseph Mercy Oakland Hospital, Pontiac, USA; 2 Neurology, Saint Joseph Mercy Oakland Hospital, Pontiac, USA

**Keywords:** cerebral vasospasm, thrombus, peripartum cardiomyopathy, stroke, reversible cerebral vasoconstriction syndrome

## Abstract

Acute cardioembolic stroke is a rare presentation of peripartum cardiomyopathy. We present an unusual case of peripartum cardiomyopathy, that subsequently developed cardioembolic ischemic stroke and reversible cerebral vasospasms.

A 26-year-old G1P1 caucasian woman presented to the emergency department 10 days after a spontaneous vaginal delivery with the clinical and physical presentation of acute heart failure. Brain natriuretic peptide (BNP) level was >8000 pg/mL. Transthoracic echocardiogram (TTE) demonstrated global left ventricular hypokinesis, reduced ejection fraction (EF) 22% with grade I diastolic dysfunction and apical thrombus. On hospital day two of her heart failure exacerbation admission, a code stroke was activated for aphasia and confusion. She received an IV tissue plasminogen activator (tPA) and underwent a mechanical thrombectomy. On hospital day three, she developed worsening of neurological symptoms, and a computed tomography (CT) angiogram revealed vasospasm in the region of the left middle cerebral artery (MCA), which subsequently resulted in nimodipine therapy. Furthermore, her hospital course was complicated by persistent hypotension, and with our concern for vasospasm that was noted in the CT angiogram instead of guideline-directed therapy for heart failure, digoxin was given to control heart rate and to improve cardiac output. Ultimately, her neurological symptoms improved, and she was discharged on hospital day 10.

This case highlights the combination of rare presentations - postpartum cardiomyopathy, ischemic stroke, and reversible cerebral vasospasms, which suggests that the time and size of the stroke are of the essence in terms of promptness of aggressive treatment.

## Introduction

Acute cardioembolic stroke is a rare presentation of peripartum cardiomyopathy. Acute ischemic stroke is three-fold higher in peripartum periods compared to non-pregnant women and is rarely reported in the literature [1, 2]. We present an unusual case of peripartum cardiomyopathy, that developed cardioembolic ischemic stroke and reversible cerebral vasospasms. 

## Case presentation

**Figure 1:**
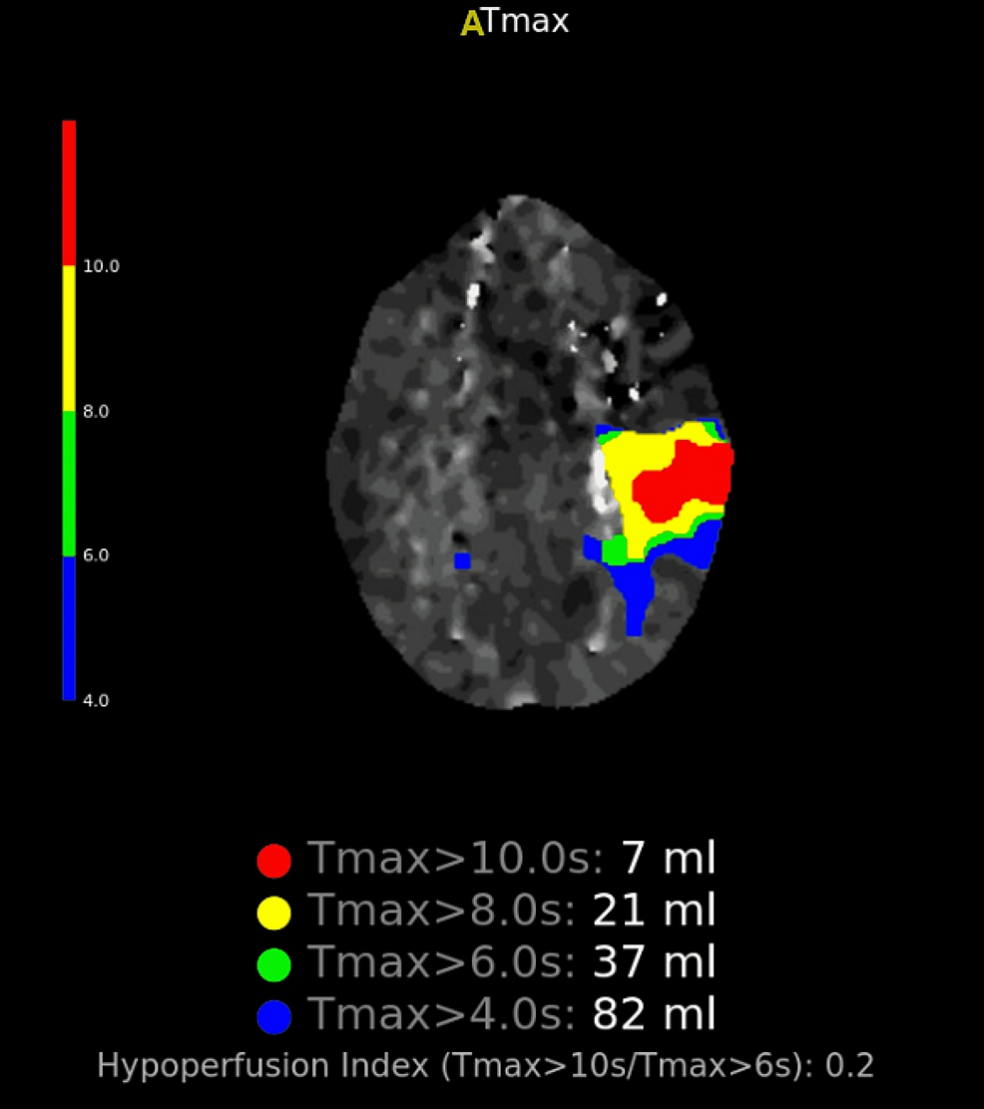
CT perfusion with contrast suggestive of acute ischemia in the left temporoparietal region. No evidence of ischemic infarct core.

**Figure 2:**
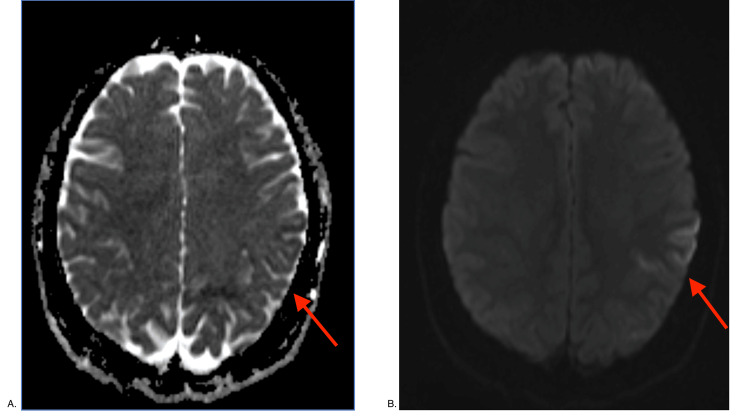
MRI of the brain without contrast (2A) that shows the restricted diffusion along the left frontoparietal cortical region, with the corresponding hypointensity on apparent diffusion coefficient (ADC), compatible with sequelae to acute ischemic infarction in diffusion-weighted imaging (DWI) in 2B (arrows).

A 26-year-old G1P1 caucasian woman with no significant past medical history presented to our facility 10 days after a spontaneous vaginal delivery with shortness of breath for three days. Vital signs included the following temperature was 36.7 degree Celsius, heart rate was 137 beats/min, oxygen saturation was 85% on room air, respiratory rate was 22 breaths/min, and blood pressure was 119/91 mmHg. Physical examination revealed pulmonary crackles, lower extremity pitting edema, and jugular venous distention. She was placed on 4 L/min of nasal cannula oxygen supplement. Laboratory evaluation was significant for brain natriuretic peptide (BNP) level was >8000 pg/mL. An electrocardiogram showed sinus tachycardia. Transthoracic echocardiogram (TTE) demonstrated global left ventricular hypokinesis and EF 22% with grade I diastolic dysfunction. She was admitted for new-onset heart failure in exacerbation. A CT angiogram of the chest was negative for pulmonary embolism with no visualization of the apical thrombus. Viral/bacterial panels, including coxsackievirus, mycoplasma IgM, and syphilis, were negative. An autoimmune panel, including antinuclear antibody, antineutrophil cytoplasmic antibodies, rheumatoid factor, Scl-70 antibody, and anti-double stranded DNA, were unremarkable. With this, the causes of cardiomyopathy were ruled out. In the subsequent days, heart failure improved clinically with aggressive diuresis with intravenous furosemide and mechanical breast pumping to induce lactation. On hospital day two, she developed expressive aphasia with confusion. Code stroke was activated. Her neurological examination fluctuated with the National Institutes of Health Stroke Scale (NIHSS), ranging between four and seven at different time periods. CT head without contrast was negative. CT angiogram of the head/neck identified a focal occlusive thrombus within the superior M2 branch of the left middle cerebral artery (MCA) with an area of penumbra in the left temporoparietal region noted in the CT perfusion study in Figure 1. She received IV tissue plasminogen activator (tPA) and underwent a successful mechanical thrombectomy within two hours. Magnetic resonance imaging (MRI) of the brain confirmed acute ischemic stroke seen in Figure 2. Post-procedure period, repeat TTE revealed a left ventricular apical thrombus. She was placed on appropriate cardiac medications (aspirin, atorvastatin, sacubitril-valsartan, and carvedilol) and a heparin drip. 

**Figure 3:**
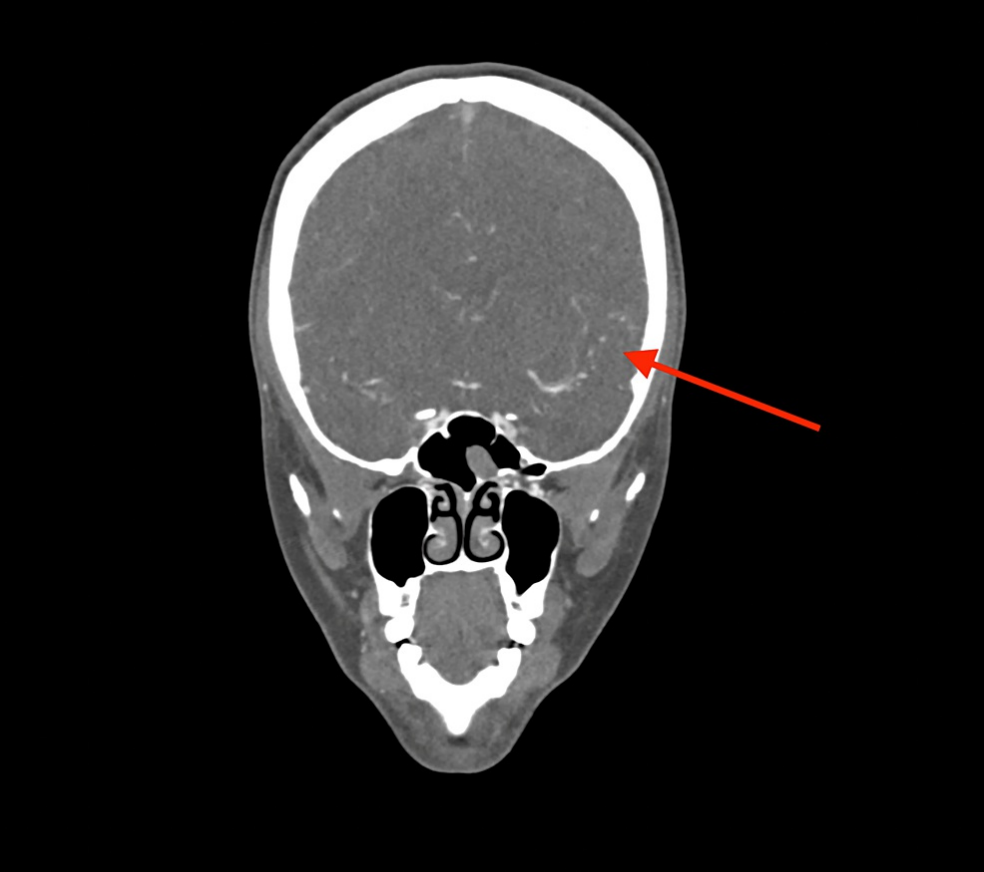
CT angiogram of the head and neck showing mild contour irregularity along the distal M1 segment of the left middle cerebral artery, suggesting underlying vasospasm (arrow).

On hospital day three, the patient complained of squiggly lines in the left eye and decreased sensation of the right upper and lower extremities with NIHSS of one. A repeat CT head without contrast was unremarkable for acute process. CT angiogram revealed vasospasm of the M1 segment of the left middle cerebral artery in Figure 3, which prompted nimodipine therapy at a dose of 60 mg every four hours. This therapy relieved her symptoms over the next 24 hours. Electroencephalogram (EEG) was negative for any epileptiform activity. As she had persistent hypotension and with our concern for vasospasm that was noted in the CT angiogram, sacubitril-valsartan and carvedilol were discontinued initially. Therefore, digoxin was given to control the heart rate as it was sinus tachycardia with a heart rate of 135 beats/min and to improve her cardiac output. The patient was successfully discharged on hospital day 10 on apixaban, nimodipine, carvedilol, and digoxin. Direct oral anticoagulant was chosen as the patient was not breast-feeding, and it was considered a non-inferior convenient treatment in comparison to warfarin or low molecular weight heparin. At the follow-up appointment after three months, she reported improvement of her symptoms. 

## Discussion

Our case is unique as the combination of peripartum cardiomyopathy, ischemic stroke, and cerebral vasospasm is rare. Peripartum cardiomyopathy is a myocardial disorder that is most common in the age group of 22-33, where the heart muscle is structurally and functionally abnormal in the absence of coronary artery disease, hypertension, valvular disease, and congenital heart disease [1-3]. The reported rate is about 1 in 3000 to 1 in 4000 live births in the United States [4, 5]. Risk factors include older age, parity, African American race, hypertension, preeclampsia, eclampsia, infection, smoking, diabetes, and cesarean delivery. [1-3]. Ischemic strokes are reported only in 5% of patients with peripartum cardiomyopathy, though the incidence of systemic thromboembolic complications is high at 25-40% [6]. Interestingly, stroke is frequently caused by cardiac emboli and rarely by cerebral hypoperfusion and vasospasms [7]. Reversible cerebral vasoconstriction syndrome (RCVS) is characterized by reversible multifocal cerebral vasospasm occurring within a week of delivery [8-10]. Our patient had fluctuating visual symptoms, like RCVS patients, with the diagnosis confirmed by CT angiography and transcranial Doppler. Though the exact etiology of RCVS in the peripartum period is unclear, sudden reduction of progesterone levels (a vasodilatory hormone) or intracerebral vasoconstriction due to decreased blood flow during delivery and puerperium period are the proposed causes [6]. In our case, cardiomyopathy could have been another contributory factor. 

Decision-making during the acute code stroke was a big dilemma. The NIHSS fluctuated between four and seven, with her becoming severely aphasic during the peak of worsening symptoms. She received tPA, and the decision to intervene with thrombectomy was made due to the evidence seen on the CT angiogram and CT perfusion that showed a large ischemic penumbra mismatch in the language area of the brain and the presence of clot in the M2 branch of the MCA. Cerebral vasospasm was noted in the CT angiogram the next day, which as a risk factor, may have caused a permanent deficit from its complications of seizure, hemorrhage, and re-infarction. 

## Conclusions

This case highlights the combination of rare presentations - postpartum cardiomyopathy, ischemic stroke, and reversible cerebral vasospasms, and the aggressive treatment for a low stroke scale in a timely manner with IV tPA and mechanical thrombectomy. These steps were key in preventing irreversible neurological damage and mortality.
